# Modifications of the Structural, Nutritional, and Allergenic Properties of Atlantic Cod Induced by Novel Thermal Glycation Treatments

**DOI:** 10.3390/foods13142175

**Published:** 2024-07-10

**Authors:** Xin Dong, Vijaya Raghavan

**Affiliations:** Department of Bioresource Engineering, Faculty of Agricultural and Environmental Sciences, McGill University, Sainte-Anne-de-Bellevue, QC H9X 3V9, Canada; vijaya.raghavan@mcgill.ca

**Keywords:** thermal glycation, Atlantic cod, microwave, protein, allergenicity

## Abstract

This study aimed to assess the effect of novel thermal glycation, utilizing microwave processing (100−150 °C) combined with sugars (glucose and lactose), on the in vitro protein digestibility, peptides, secondary structures, microstructures, and allergenic properties of Atlantic cod. The research demonstrated that microwave heating at 150 °C with glucose significantly reduced cod allergenicity by up to 16.16%, while also enhancing in vitro protein digestibility to 69.05%. Glucose was found to be more effective than lactose in conjunction with microwave heating in reducing the allergenicity of Atlantic cod. Moreover, treatments conducted at 150 °C were more effective in increasing in vitro protein digestibility and peptide content compared to those at 100 °C. This study revealed that the novel processing technique of thermal glycation effectively reduced the allergenicity of Atlantic cod. It also offered fresh insights into the potential benefits of combining microwave heating with sugars.

## 1. Introduction

Fish serves as an important source of protein in human diets worldwide. However, the allergenicity of fish products has become a growing concern, with reports of adverse reactions to fish consumption increasing. In particular, Atlantic cod has been identified as a common cause of fish allergies [1]. Parvalbumin is the major fish allergen commonly found in fish muscle tissue, which belongs to the calcium-binding protein family with stable, heat-resistant, and cross-reactivity properties [2,3]. However, effective food processing techniques can lead to reduced allergenicity of parvalbumin by altering its structure.

Microwave heating is regarded as a novel thermal processing method. In comparison to conventional thermal techniques (e.g., boiling and steaming), microwave heating shows many advantages, such as eco-friendliness, cost-efficiency, rapid and uniform heat distribution, and user-friendly operation [4]. Microwave heating has the potential to modify the native conformation of proteins, possibly influencing their recognition by IgE in individuals with allergies. Additionally, this method exerts a minimal negative effect on the flavor and nutritional integrity of food products throughout processing [5].

Thermal glycation is a chemical reaction between reducing sugars and proteins, leading to the formation of advanced glycation end-products (AGEs) [6]. AGEs have been associated with a range of physiological consequences, including the exacerbation of allergic reactions. In comparison to conventional thermal methods, microwave promotes glycation in food proteins by changing protein secondary and tertiary structures [7]. In addition, microwave heating with glycation improves food proteins’ properties and increases the interactions more than conventional water-bath heating [8].

Wu, et al [9] reported that thermal glycation may result in changes to the structure of proteins and decreases the nutritional value of food products. Zhao, Cai [10] reported that thermal glycation treatment (glucose at 60 °C for 72 h) led to a reduction in IgE binding in recombinant silver carp parvalbumin detected by dot blot analysis. However, existing studies have only used conventional thermal processing in conjunction with glycation, and few studies have focused on Atlantic cod treated by thermal glycation. In this study, novel thermal glycation treatment was applied by combining microwave (100−150 °C) and sugars (glucose and lactose) to investigate the impact on the allergenic properties of Atlantic cod. Furthermore, this study evaluated alterations in protein secondary structures, microstructures, peptides, and the in vitro protein digestibility of Atlantic cod during thermal glycation treatments. This study is the first to discuss microwave heating with glycation applied to Atlantic cod, offering a novel approach that could improve food safety and nutritional quality by reducing allergenic properties and enhancing protein digestibility.

## 2. Materials and Methods

### 2.1. Sample Preparation

Fresh Atlantic cod meat was obtained from L’artisan de La Mer market located in Montreal, Canada. Cod filets were cut into pieces and thawed to room temperature. The filets were ground and homogenized with a knife mill GRINDOMIX GM 200 (4000 RPM for 90 s, 1000W, Retsch, Newtown, PA, USA) into minced cod. Glucose (D-Glucose, Anhydrous, Granular Powder/Certified ACS, Fisher Scientific, Singapore) and lactose (α-D-Lactose monohydrate, ACS, Fisher Scientific, Singapore) solutions were prepared for 2 mg/mL by using distilled water. The minced cod was treated by mixing with glucose and lactose solutions by a ratio of 1:1 (per gram/per milliliter) in the glass tube. A Mini WAVE Digestion Module (SCP Science, 115 V, 60 Hz, 15 A, 1000 W, Montreal, QC, Canada) was used at 100 and 150 °C for 10 min. The thermal glycation treatments were set as (1) control, (2) MW100, (3) MW150, (4) MW-LAC100, (5) MW-LAC150, (6) MW-GLU100, and (7) MW-GLU150, respectively (Table 1). The processed samples were subjected to a freeze-dryer (7420020, Labconco Corporation, Kansas City, MO, USA) for 48 h. The cod powder was stored at −20 °C until further analysis. All treatments and analyses were performed in triplicates.

### 2.2. Total Soluble Protein Content

Total soluble protein content was determined by extracting freeze-dried cod powder (0.2 g) with phosphate-buffered saline (PBS) solution (0.1 M, pH = 7). Following 30 min incubation at room temperature, the mixture was centrifuged at 5000× *g*, 4 °C for 20 min. The supernatant was collected for subsequent analysis. The total soluble protein content in cod samples was determined using a Pierce™ BCA protein assay kit (Thermo Fisher Scientific, Mississauga, ON, Canada) following the provided protocol. The results are reported as micrograms of soluble protein per gram of freeze-dried cod powder (mg/g).

### 2.3. Peptide Content

The peptide content was determined using o-Phthaldialdehyde (OPA) reagent. The preparation of the OPA reagent followed the protocol mentioned by Dong and Raghavan [11]. The 190 μL of OPA reagent was mixed with the 10 μL of digestion mixture for a 2 min incubation. The final absorbances were read by a plate reader (Synergy HTX Multi-Mode Reader, BioTek Instruments, Toronto, ON, Canada). The results were recorded at 340 nm with leucine–glycine for the standard curve.

### 2.4. In Vitro Protein Digestibility

The in vitro digestibility of cod proteins was measured in two stages based on the previous procedure motioned by Dong, Wang [4]. A mixture (20 mg/mL) of freeze-dried cod powder (0.2 g) and a PBS buffer (0.1 M, 10 mL, pH = 7.4) was prepared. The protein content before digestion and after every stage of digestion was used for the calculation of in vitro protein digestibility (IVPD%) according to the following equation:

(1) $$IVPD\%=\frac{protein\;content\;before\;digestion-protein\;content\;after\;digestion}{protein\;content\;before\;digestion}\times 100.$$

### 2.5. FTIR Analysis

Fourier transform infrared spectroscopy (Nicolet Magna 158,750 FTIR, Nicolet Instrument Corp., Madison, WI, USA) was used to investigate secondary structures in cod protein. The absorbance was scanned in the spectra region (1200–1800 cm^−1^) with a resolution of 4 cm^−1^ by OMNIC software (Version 8, Thermo Nicolet Co., Madison, WI, USA). The software OriginPro 2022 was used to fit secondary structural peaks in the amide I band (1700–1600 cm^−1^). During the peak-fitting progress, the second derivative spectrum was used to identify the component bands. Following the appropriate wavelength and peak areas obtained, the proportion of each secondary structure in cod proteins can be estimated.

### 2.6. CD Spectra Analysis

Circular dichroism (CD) spectroscopy was used to examine the secondary structures of cod protein. Circular dichroic measurements were determined by Chirascan (Applied Photophysic, Beverly, MA, USA) with the thermostat set at 23.4 °C. Each sample was prepared with PBS to a concentration of 6 mg/mL and filtered through a 0.22 μM membrane. All samples were scanned from 190 to 260 nm with a bandwidth of 1 nm, time per point used was 0.5 s, and the scanning time was 65 s. A cuvette with a path length of 1 mm was filled by 185 uL of sample for each sample measurement. Five accumulated spectra averaged the final spectrogram. The DichroWeb (http://dichroweb.cryst.bbk.ac.uk, accessed on 6 November 2023) was used to analyze protein secondary structures.

### 2.7. SEM Observation

A Scanning Electron Microscope (SEM) (TM3000, Hitachi High-Technologies Corporation., Tokyo, Japan) was applied to observe microstructural changes in cod samples. A piece of freeze-dried cod sample was transferred to the measuring platform for observation. For microstructural visualization, the images were captured when they were magnified 100 times.

### 2.8. SDS-PAGE Analysis

Sodium dodecyl sulfate–polyacrylamide gel electrophoresis (SDS-PAGE) was performed according to the following protocol. The protein extract (10 μL) was mixed with β-mercaptoethanol and 2 × Laemmli sample buffer and then heated at 95 °C for 5 min. Running buffer was prepared by 10× Tris/Glycine/SDS mixed with distilled water. Samples were loaded for 10 μL per lane, and a molecular weight marker (10–250 kDa) (Bio-Rad, Philadelphia, PA, USA) was loaded for 5 μL. Electrophoresis was performed in a vertical unit (Mini-PROTEAN^®^ Tetra System, BIO-RAD, Philadelphia, PA, USA) at 200 V. After electrophoresis, gels were washed in water for 5 min. After removing the water, the gel was stained by Bio-Safe^TM^ Coomassie G-250 Stain (Bio-Rad, Hercules, CA, USA) for 1 h and then was rinsed with water for 30 min. The image was captured using a digital camera (Canon, EOS Rebel SL2 DSLR Camera, Newport News, VA, USA).

### 2.9. ELISA Analysis

A sandwich ELISA (Fish Parvalbumin ELISA Kit, Arigo Biolaboratories, Shanghai, China) was utilized to quantify the parvalbumin content in the cod samples according to protocol. After incubation and washing several times following microplate procedures, the solution color changed from blue to yellow. The absorbances were read at 450 nm by a microplate reader (Synergy HTX Multi-Mode Reader, BioTek Instruments, Toronto, ON, Canada).

### 2.10. Statistical Analysis

The analysis of variance (ANOVA) feature of the SPSS program (IBM SPSS Statistic, Ver. 29.0.0.0) was used to examine the experimental data. The means were separated using the Duncan multiple range test, and significance was established at *p* ≤ 0.05.

## 3. Results and Discussions

### 3.1. Protein and Peptide Content

**Figure 1:**
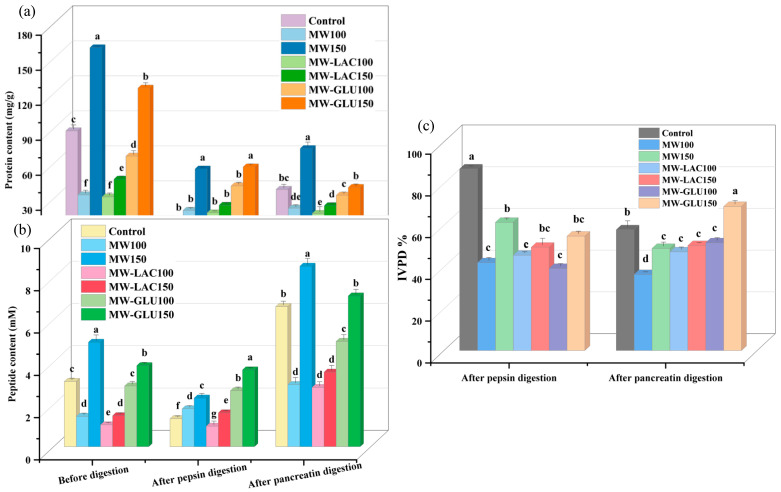
The protein content (**a**) and peptide content (**b**) of cod samples before and after each stage of digestion, and the IVPD percentage (**c**) after two-stage digestion. Note: the different letters at the top of each bar indicate the significant difference (*p* < 0.05) among each treatment.

The total soluble protein content of cod samples was determined (Figure 1a). Compared to the control (86.84 mg/g), the soluble protein content of all samples treated by thermal glycation could be significantly increased. The highest soluble protein content before digestion was observed in the MW150 (158.04 mg/g) and MW-GLU150 (123.42 mg/g), while the lowest was in the MW-LAC100 (30.17 mg/g) and MW100 (31.96 mg/g). This difference in soluble protein content is probably due to the temperature changes in thermal processing and the variations of protein solubility in different treatments [12,13]. After pepsin digestion, an increase in protein content was observed in all treated samples compared to the control (11.28 mg/g). The highest protein content was still found in the MW-GLU150 (55.88 mg/g) and MW150 (54.13 mg/g) with a significant difference, while other samples showed an increase without a significant difference. The pancreatin digestion led to different results compared to the pepsin digestion, with some treatments (MW100, MW150, and control) showing a higher protein content after pancreatin than after pepsin digestion. The increased protein contents were probably because of protein aggregation at higher pH values [14]. Following pancreatin digestion, the highest protein content was again in the MW150 sample (71.66 mg/g) with a significant difference, and the lowest was in the MW-LAC100 sample (16.03 mg/g). The results indicated that thermal glycation treatments, especially using microwaves at higher temperatures, significantly impact the protein content in cod. The MW treatment at 150 °C consistently resulted in the highest protein content before or after digestion, which suggests that this treatment might help in retaining or making more proteins soluble. There is a noticeable difference in protein content post-pepsin and post-pancreatin digestions. This suggests that different digestive enzymes interact uniquely with the protein structure altered by thermal treatments, impacting the solubility and breakdown of proteins. The inclusion of glucose and lactose in the treatment (especially at 100 °C) generally resulted in lower protein content compared to the highest results (MW at 150 °C). This could be due to the Maillard reaction between sugars and proteins, affecting protein structure and solubility [15]. The higher solubility of proteins post certain treatments at higher temperatures (like MW at 150 °C) could be beneficial for dietary purposes and in the food industry.

For the peptide content before the digestion, compared with the control (3.09 mM), two treated samples including MW150 (4.94 mM) and MW-GLU150 (3.84 mM) showed a significant increase, whereas others significantly decreased (Figure 1b). The lowest peptide content was found in the MW-LAC100 sample (1.03 mM). In comparison to the pre-digestion levels, pepsin digestion appeared to reduce peptide content significantly in most samples, except for the MW100 and MW-LAC150 samples, which increased slightly. After pepsin digestion, the highest peptide content was found in the MW-GLU150 sample (3.64 mM), and the lowest in the MW-LAC100 sample (0.97 mM). Afterwards, the pancreatin digestion resulted in a general increase in peptide content compared to both pre-digestion and post-pepsin digestion levels for all samples. Following pancreatin digestion, the highest peptide content was in the MW150 sample (8.54 mM) and MW-GLU150 sample (7.13 mM) with a significant difference, and the lowest was in the MW-LAC100 and MW100 samples (around 2.80 mM). The MW treatment at 150 °C consistently resulted in the highest peptide content, particularly after pancreatin digestion. This suggests that microwave treatment at higher temperatures might be more effective in preserving or enhancing peptide solubility and availability. The different impacts of pepsin and pancreatin digestions on peptide content indicate that these enzymes have distinct modes of action on the protein–peptide matrix, especially after thermal treatments [15]. The addition of sugars in the treatment process (especially lactose at 100 °C) generally resulted in lower peptide content compared to the highest results. This could imply that sugars may interact with protein structures in a way that affects peptide release or solubility [16]. The increased peptides after digestion in the high-temperature microwaved treatment could improve the bioavailability of peptides and potential health benefits.

### 3.2. In Vitro Protein Digestibility (IVPD)

The IVPD results of cod samples with thermal glycation treatments are shown in Figure 1c. After the first stage (pepsin) of digestion, the treated samples showed an overall decreased IVPD compared with that of the control (87.05%). The highest and the lowest IVPD percentages were in the MW150 sample (61.45%) and the MW-GLU100 sample (39.37%), respectively. After pancreatin digestion, the IVPD values of most treated samples showed a decreasing trend except for the MW-GLU150 sample. The final IVPD percentage was highest for the MW-GLU150 sample (69.05%) with an increase of 19.26% in comparison to the control (57.90%). The MW100 treatment resulted in the lowest final IVPD percentage (36.45%). These results indicated a significant impact of thermal glycation treatments on the digestibility of proteins in cod. The MW treatment at 150 °C and the addition of glucose at the same temperature generally maintained or improved digestibility, while the MW treatment at 100 °C significantly reduced it. Moreover, there is a notable difference in IVPD percentages between pepsin and pancreatin digestions. This suggests that these enzymes have different efficiencies in digesting the modified protein structures resulting from the various thermal treatments. The addition of sugars, particularly glucose at 150 °C, seems to enhance the digestibility of proteins post-treatment, which could be attributed to alterations in protein structure that make them more susceptible to enzymatic breakdown [17]. Therefore, thermal glycation treatments using microwaves at higher temperatures and glucose significantly influence the digestibility of cod proteins. This provided suggestions for designing processing methods to optimize the nutritional value of cod. Conversely, the IVPD values of whey protein α-lactalbumin decreased from 25.91% to 10.93% with increasing temperature (60−100 °C) [18]. Because protein digestion and absorption are important for human health, an increase in protein digestibility results in an enhancement of protein nutrition and could be beneficial to the human body.

### 3.3. FTIR and CD Spectra Analysis

**Figure 2:**
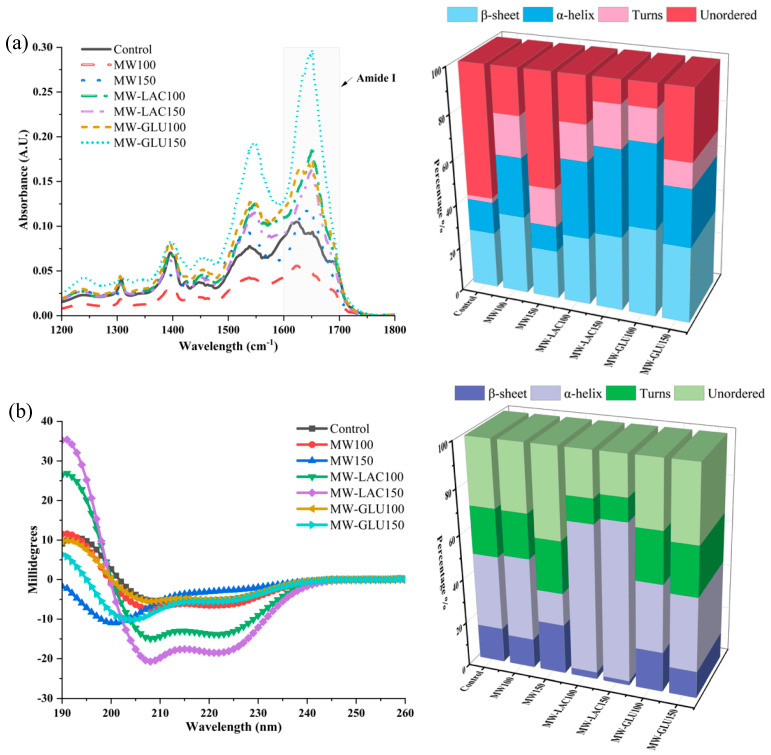
FTIR spectra and the percentage changes in protein secondary structure by FTIR spectra (**a**); CD spectra and the percentage changes in protein secondary structure by CD spectra (**b**).

The impact of different thermal glycation treatments on the protein secondary structure of cod was evaluated by FTIR and CD spectra. In Figure 2a, the absorbance values vary significantly across the different treatments. The MW-GLU150 treatment showed a notable difference in absorbance, which might indicate significant protein structural changes. Furthermore, the FTIR analysis also revealed significant alterations in the percentage of protein secondary structure. Compared with the control, MW100 showed a notable increase in β-sheet (35.38%) and α-helix (26.54%) contents. In MW150, there was a similar β-sheet content to the control but a decrease in α-helix and unordered structures. MW-LAC and MW-GLU Treatments showed a remarkable increase in β-sheet content, especially in MW-GLU100 (39.20%), indicating a profound impact of glucose in promoting β-sheet formation. The FTIR results demonstrate that thermal glycation treatments can significantly alter the secondary structure of proteins in cod. These changes could be due to the Maillard reaction between proteins and sugars or heat-induced denaturation [19]. The alterations in the amide I and II bands suggest changes from α-helices to β-sheets, which are often associated with protein aggregation or denaturation. Alterations in protein structure could also influence the digestibility and allergenicity of the proteins [20].

The CD spectroscopy results provide insights into the secondary structure of cod protein. The data show variations in CD spectra across treatments, indicating alterations in the protein’s secondary structure due to the thermal glycation process (Figure 2b). For the percentage changes, in comparison to the control, MW100 demonstrated a slight decrease in β-sheets (13.3%) and an increase in α-helices (37.3%), whereas MW150 showed an increase in β-sheets (22.2%) and a decrease in α-helices (14.4%). In MW-LAC100 and MW-LAC150, a marked increase in α-helices (67.5% and 70.4%, respectively) with reduced β-sheet content was observed. Moderate changes occurred with increased β-sheet content in MW-GLU100 (17.7%) and balanced β-sheets (12.0%) and α-helices (33.4%) in MW-GLU150. Higher temperatures and adding sugars could lead to more pronounced structural changes, possibly due to enhanced Maillard reactions and heat-induced denaturation. This is evident from the distinct CD profiles of samples treated with MW-GLU and MW-LAC at 150 °C compared to the control. Similarly, Ma, Chen [21] found that increasing amounts of the α-helix and β-sheet in egg white ovalbumin were observed after 30 min glycation by glucose. Wu, Dong [9] revealed that the secondary structure of glycated β-conglycinin gradually changed from α-helix and β-sheet structures to random coil structures with increasing thermal processing degree (100−180 °C).

FTIR spectroscopy and CD spectroscopy provided different estimates of protein secondary structures due to their distinct measurement techniques and sensitivities. FTIR spectroscopy has been recognized for its sensitivity to the secondary structure of proteins, particularly its ability to detect β-sheet structures due to the distinctive absorption patterns in the amide I region (1600–1700 cm^−1^) [22]. As for CD spectroscopy, it is generally more sensitive to α-helical content because α-helices produce a strong dichroic signal in the far-UV region (200−250 nm) [23]. Additionally, CD spectroscopy typically analyzes liquid samples, while FTIR spectroscopy analyzes solid samples. Environmental factors such as pH, ionic strength, and solvent composition can also influence the results [24].

### 3.4. ELISA Analysis and SDS-PAGE

**Figure 3:**
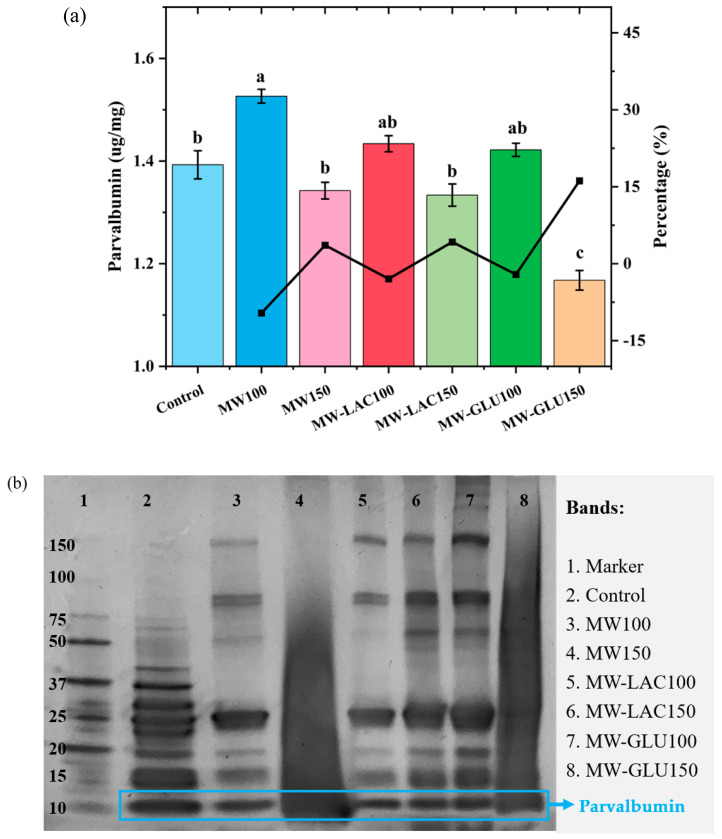
ELISA analysis of parvalbumin content (**a**) and SDS-PAGE result (**b**) cod samples before and after thermal glycation treatment. Note: the different letters at the top of each bar indicate the significant difference (*p* < 0.05) among each treatment.

ELISA analysis was used to quantify the major cod allergen parvalbumin following various thermal glycation treatments (Figure 3a). The allergenic capacity showed an overall increase at 100 °C whereas a decrease at 150 °C. This is because heating induces protein unfolding to expose epitopes, which further causes higher allergenicity to be determined [25]. The allergenicity in MW100 showed a significant increase (9.60%). In MW-LAC100 and MW-GLU100, the protein allergenicity increased slightly with no significant difference compared with the control. MW150 and MW-LAC150 showed a slightly lower allergenicity compared to the control but no significant change in the percentage. MW-GLU150 showed the lowest parvalbumin content with a significant reduction in allergenicity percentage (16.16%). The ELISA results indicate that thermal glycation treatments can modulate the allergenicity of cod. Thermal processing masks some of the linear epitopes due to protein aggregation to lower allergenicity, but it also unfolds protein to expose conformational epitopes to increase allergenicity [25]. The presence of glucose at 150 °C (MW-GLU150) could further decrease the allergenic potential. This could be attributed to the formation of advanced glycation end-products (AGEs) that may affect the immunoreactivity of parvalbumin [26]. However, lactose and glucose at 100 °C could slightly increase cod allergenicity without significant change. Similarly, Gruber and Becker [27] found that heating (100 °C for 90 min) with sugars (glucose, maltose, or ribose) led to a slightly increased allergenicity in peanuts. Ma, Chen [21] revealed that heating and glucose together could significantly reduce the allergenicity of egg white ovalbumin, especially with a longer heating time (30−60 min). Allergenicity changes occurred during the Maillard reaction. The protection of the epitopes by the covalent attachment of reducing sugar chains may be the mechanism behind this. Additionally, Maillard reactions could change the allergen structure by exposing hidden interior epitopes to bind IgE antibodies or destroying conformational epitopes [21].

SDS-PAGE analysis was conducted to determine the protein profiles of cod samples with various thermal glycation treatments (Figure 3b). For all bands, Lane 3 (MW100) and Lane 5 (MW-LAC100) showed a slightly altered banding pattern from the control, indicating changes in protein structure or degradation. Lane 4 (MW150) and Lane 8 (MW-GLU150) exhibited diffuse banding patterns with clear smearing. They were the most destroyed bands with further alterations in the protein profile, suggesting more pronounced thermal effects at a higher temperature (150 °C) leading to structural degradation [28]. Lane 6 (MW-LAC150) and Lane 7 (MW-GLU100) showed similar band intensity and the possible presence of new bands, possibly due to Maillard reaction products and sugars added [15]. For the band of parvalbumin, the band intensity was changed in all treated samples (Lane 3 to 8) compared to the control (Lane 2). Lane 5 (MW-LAC100) showed the enhanced intensity of parvalbumin visually and probably caused the increased allergenicity, which was in correspondence with the ELISA results. Although other changes in the parvalbumin band were not obvious, the SDS-PAGE results revealed that microwave heating (especially at 150 °C) led to protein modifications including unfolding, aggregation, or fragmentation [29]. These changes are more noticeable at higher temperatures, as evidenced by the diminished band intensities in Lane 4 (MW150) and Lane 8 (MW-GLU150). Thus, thermal glycation treatments at higher temperatures may reduce the allergenicity of cod by decreasing parvalbumin.

### 3.5. SEM Observation

**Figure 4:**
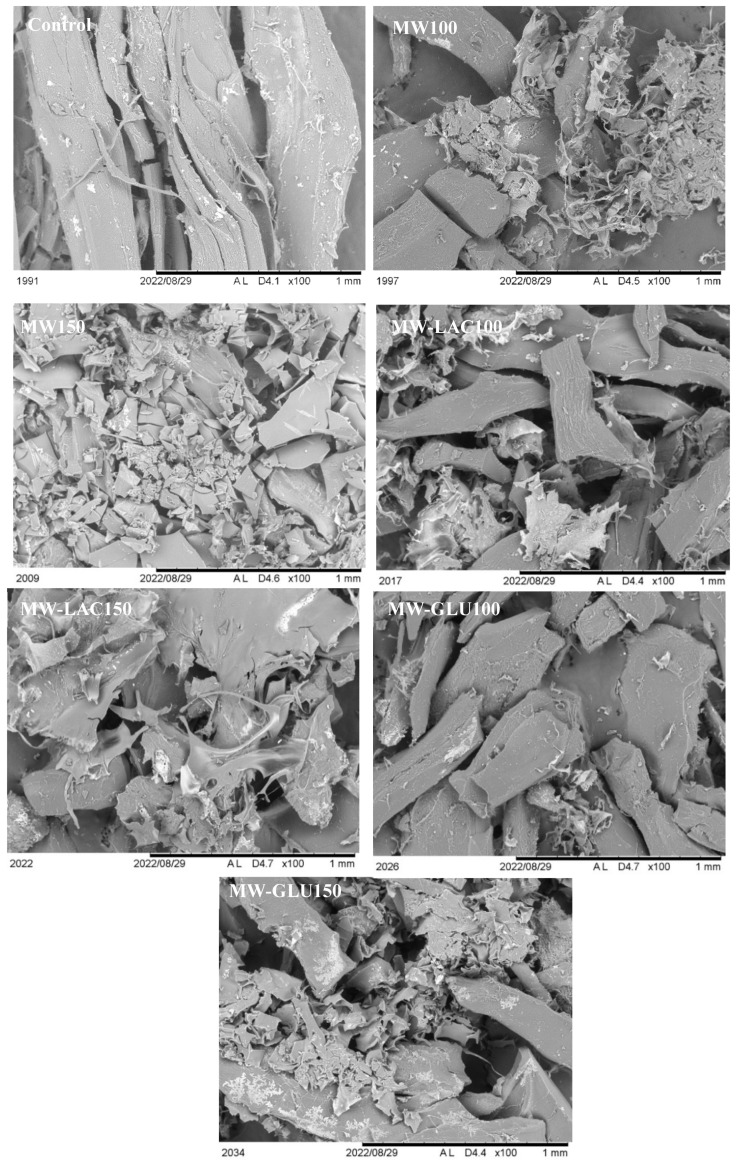
SEM images of cod samples treated by thermal glycation (magnification of 100 times).

The microstructural changes in the samples were observed by SEM at 100 magnifications (Figure 4). After treatments, the apparent structure of the proteins was altered from block to broken and the protein surfaces turned from smooth to rough. Additionally, protein aggregations and protein folding were noted, which were probably due to the modifications of the second and tertiary structures of proteins [30]. Thus, microstructural changes were related to structural changes and may further affect allergenicity. Similar results were also observed in other research. Pi, Sun [31] reported that the microstructure changed with the holes and rough surfaces generated in soybeans after boiling (100 °C) and autoclaving (121 °C) for 20 min.

## 4. Conclusions

In conclusion, the study revealed significant insights into the impact of thermal glycation on the physicochemical and allergenic properties of Atlantic cod. It showed that applying microwave heating at 150 °C along with glucose markedly decreased cod’s allergenicity by as much as 16.16% and simultaneously improved in vitro protein digestibility to 69.05%. It was observed that glucose, when used with microwave heating, outperformed lactose in reducing the allergenicity of Atlantic cod. Furthermore, treatments at 150 °C proved to be superior in enhancing both in vitro protein digestibility and peptide content when compared to treatments at 100 °C. These findings contribute to the scientific understanding of protein allergenicity and physicochemical properties in the context of food processing. Additionally, they have practical implications in developing processing methods aimed at improving the management of allergenic foods, enhancing food safety for consumption by a broader population.

## Figures and Tables

**Table 1:** Experimental setup of thermal glycation treatments for cod samples.

Treatments	Thermal Treatment	Temperature (°C)	Sugars	Duration (min)
Control	−	RT	−	10
MW100	Microwave	100	−	10
MW150	Microwave	150	−	10
MW-LAC100	Microwave	100	Lactose	10
MW-LAC150	Microwave	150	Lactose	10
MW-GLU100	Microwave	100	Glucose	10
MW-GLU150	Microwave	150	Glucose	10

Note: RT is the abbreviation of room temperature.

## Data Availability

The original contributions presented in the study are included in the article, further inquiries can be directed to the corresponding author.
